# Epidemiology, Species Susceptibility, and Mortality Risk Factors of Pediatric Candidemia: A 13-Year Multicenter Study

**DOI:** 10.3390/jof12090695

**Published:** 2026-09-16

**Authors:** Elias Nasrallah, Avi Magid, Michal Stein, Elina Gelman, Dana Danino, Lital Hadad, Liat Ashkenazi-Hoffnung, Meirav Mor, Nimrod Sachs, Naama Shechter, Galia Grisaru-Soen, Asaf Regev, Diana Tasher, Amir Weintraub, Orli Megged, Alex Guri, Roni Gur Lavy, Or Kriger, Ronen Ben-Ami, Yael Shachor-Meyouhas

**Affiliations:** 1 Pediatric Infectious Diseases Unit, Rappaport Children’s Hospital, Rambam Health Care Campus, P.O. Box 9602, Haifa 3109601, Israel; y_shahor@rambam.health.gov.il; 2 The Azrieli Faculty of Medicine, Bar-Ilan University, Safed 1311502, Israel; 3 Hospital Management, Rambam Health Care Campus, Haifa 3109601, Israel; magid.avi@gmail.com; 4 Department of International Health, Maastricht University, P.O. Box 616, 6200MD Maastricht, The Netherlands; 5 School of Public Health, Ben-Gurion University, Be’er Sheva 8410501, Israel; 6 Pediatric Infectious Disease Unit, The Edmond and Lily Safra Children’s Hospital, Chaim Sheba Medical Centre, Ramat Gan 52621, Israel; michal.stein@sheba.health.gov.il (M.S.); elina.gelman@sheba.health.gov.il (E.G.); orkriger@gmail.com (O.K.); 7 Gray Faculty of Medical & Health Sciences, Tel Aviv University, Tel Aviv 69978, Israel; liat.ashkenazi@clalit.org.il (L.A.-H.); meiravm@clalit.org.il (M.M.); nimrod_rod@yahoo.com (N.S.); galiag@tauex.tau.ac.il (G.G.-S.); assafre84@gmail.com (A.R.); dtasher@gmail.com (D.T.); amir.weintraub@gmail.com (A.W.); ronen.benami@gmail.com (R.B.-A.); 8 Pediatric Department A, Saban Children’s Hospital, Soroka University Medical Center, Beer-Sheva 84101, Israel; danadanino@hotmail.com (D.D.); litalhadad6@gmail.com (L.H.); 9 The Faculty of Health Sciences, Ben-Gurion University of the Negev, Beer-Sheva 8410501, Israel; 10 Pediatric Infectious Disease Unit, Schneider Children’s Medical Center of Israel, Petah Tikva 4920235, Israel; 11 Department of Day Hospitalization, Schneider Children’s Medical Center, Petah Tikva 4920235, Israel; namana@clalit.org.il; 12 Infection Control Unit, Schneider Children’s Medical Center of Israel, Petah Tikva 4920235, Israel; 13 Pediatric Infectious Disease Unit, Dana-Dwek Children’s Hospital, Tel Aviv Sourasky Medical Center, Tel Aviv 6423906, Israel; 14 Pediatric Infectious Disease Unit, E. Wolfson Medical Center, Holon 5822012, Israel; 15 Pediatric Department, E. Wolfson Medical Center, Holon 5822012, Israel; 16 Pediatric Department, Shaare Zadek Medical Center, Jerusalem 9103102, Israel; orlimegged@yahoo.com; 17 Faculty of Medicine, Hebrew University of Jerusalem, Jerusalem 9112102, Israel; alexgur@clalit.org.il; 18 Pediatric Infectious Diseases, Kaplan Medical Center, Rehovot 7661041, Israel; 19 Pediatric Infectious Diseases Unit, Shamir Medical Center, Zerifin 7033001, Israel; ronni.gl@gmail.com; 20 Herta and Paul Amir School of Medicine, University of Haifa, Haifa 3498838, Israel; 21 Infectious Diseases Unit, Tel Aviv Sourasky Medical Center, Tel Aviv 6423906, Israel; 22 The Ruth & Bruce Rappaport Faculty of Medicine, Technion-Israel Institute of Technology, Haifa 3525433, Israel

**Keywords:** candidemia, mortality, pediatric, *Candida albicans*, non-*albicans Candida*, antifungal susceptibility, fluconazole resistance

## Abstract

*Candida* species are the leading cause of invasive fungal infections in children and are associated with substantial morbidity and mortality. We conducted a multicenter retrospective study across nine hospitals in Israel, including all candidemia episodes in patients aged 0–18 years between 2010 and 2022. Clinical, microbiological, and outcome data were collected, and multivariable logistic regression was used to identify predictors of 30-day mortality. A total of 498 candidemia episodes were identified in 482 patients. Most episodes occurred in immunocompromised children or those admitted to intensive care units. Non-*albicans Candida* species predominated (63.3%), although *C. albicans* remained the single most common species (36.7%). The most frequent non-*albicans* species were *C. parapsilosis* (25.5%), *C. tropicalis* (15.9%), and *C. glabrata* (9.6%). No cases of *C. auris* were identified. Fluconazole non-susceptibility was observed in 19.4% of isolates and increased from 15.5% during 2010–2016 to 23.3% during 2017–2022 (OR 2.89, 95% CI 1.45–5.11, *p* = 0.007). The 30-day mortality rate was 23.4%. Independent predictors of mortality included liver disease, neutropenia, total parenteral nutrition, urinary tract infection, skin lesions, and peritonitis. Pediatric candidemia remains associated with considerable mortality, while increasing fluconazole non-susceptibility among non-*albicans Candida* species highlights the need for ongoing surveillance and antifungal stewardship.

## 1. Introduction

Candidemia is the third leading cause of nosocomial bloodstream infections in children in the United States and Europe, associated with high rates of morbidity and mortality [1,2]. Rates of candidemia are increasing in adults and children, mostly reflecting the prolonged survival of complicated and immunocompromised patients resulting from advances in medical therapies [3,4]. The overall reported mortality of candidemia in children ranges between 10 and 38 percent [1,5].

The distribution of *Candida* species causing invasive infection varies geographically and over time. While *Candida albicans* remains the most frequently isolated species, non-*albicans* species are increasingly isolated, with *Candida parapsilosis* emerging as the predominant non-*albicans* species among children in many geographic regions [1,6]. Over the last decade, *Candidozyma auris* has emerged as a global health threat because of its rapid spread and multidrug resistance, making it a difficult-to-treat nosocomial pathogen. Outbreaks in adults with relatively high mortality were described, while among pediatric patients there are some reports with rising concern in this population [7,8].

Risk factors for 30-day mortality among pediatric patients in previous studies included hospitalization in a pediatric intensive care unit (PICU), exposure to systemic steroids, previous treatment with azoles or carbapenems, male sex, liver disease, presence of shock, and mucositis [6,9,10].

We aimed to describe the epidemiology, clinical characteristics, and susceptibility of *candida* species, as well as mortality risk factors of candidemia among pediatric patients in Israel.

## 2. Methods

A multicenter retrospective study that included all candidemia episodes in pediatric patients aged 0–18 years that occurred between 1 January 2010, and 31 December 2022. Nine hospitals in Israel participated in the study; five were tertiary care centers (out of 6 tertiary centers in Israel).

Both neonatal and non-neonatal patients were included. Isolates were collected from all clinical services rather than from selected hospital areas. 

Episodes were included if *Candida* spp. was isolated from at least one blood culture; consistent with current practice, any *Candida* isolated from blood was considered clinically significant. If *Candida* spp. were isolated from more than one blood culture, the episode was considered a separate event if a different *Candida* species was identified ≥48 h apart, or if the same species was recovered ≥30 days after the first negative blood culture and was considered a new episode following review of the clinical and microbiological data by a pediatric infectious diseases specialist. Candidemia duration was calculated from the date of the first positive blood culture taken to the date of documented clearance.

Data were collected from medical records and included personal and demographic variables, clinical and microbiological characteristics, outcomes (including 30-day mortality), and complications.

Diagnosis of *Candida* organ involvement was established according to the IDSA guidelines [11]. Central line-associated blood stream infection (CLABSI) was defined according to the Centers for Disease Control and Prevention (CDC) surveillance criteria [12].

Twenty-four isolates of *Candida krusei* (*Pichia kudriavzevii*) were classified as fluconazole-resistant, as this species is intrinsically resistant to fluconazole and the CLSI does not provide fluconazole susceptibility breakpoints for *C. krusei* [13].

For species–antifungal combinations for which CLSI clinical breakpoints were unavailable, epidemiological cutoff values were not applied, and these isolates were excluded from the corresponding susceptibility/resistance analyses.

### 2.1. Microbiological Identification and Antifungal Susceptibility Testing

*Candida* isolates recovered from blood cultures were identified according to the routine diagnostic procedures in use at each participating laboratory during the study period. Yeast identification was primarily performed using the VITEK 2 system with the YST ID card (bioMérieux, Marcy l’Étoile, France), supplemented, when applicable, by presumptive identification based on colony morphology on CHROMagar *Candida* chromogenic medium (BD Life Sciences, Franklin Lakes, NJ, USA, or HyLabs, Rehovot, Israel). Isolates were routinely identified by matrix-assisted laser desorption/ionization time-of-flight mass spectrometry (MALDI-TOF MS) using either the VITEK MS system (bioMérieux, Marcy l’Étoile, France) or the MALDI Biotyper system (Bruker Daltonics GmbH & Co. KG, Bremen, Germany). In the rare cases in which routine identification was inconclusive or additional fungal identification was required, panfungal PCR targeting the 28S rRNA gene followed by sequencing was performed on cultured isolates.

Antifungal susceptibility testing methods varied over the study period. Initially, susceptibility testing was primarily performed using ETEST gradient diffusion strips (bioMérieux, Marcy l’Étoile, France); subsequently, testing was primarily performed using the Sensititre YeastOne system (Thermo Fisher Scientific, Waltham, MA, USA). In selected cases, the VITEK 2 system with the AST-YS08 card (bioMérieux, Marcy l’Étoile, France) was used. These methods provided minimum inhibitory concentration (MIC) values for the antifungal agents tested, which were interpreted according to Clinical and Laboratory Standards Institute (CLSI) breakpoints [13,14].

Quality-control procedures were performed using standard reference strains, including *Candida albicans* ATCC 14053, *Candida parapsilosis* ATCC 22019, and *Candida krusei* ATCC 6258.

Since there are several participating hospitals, there might be a difference with the systems and the years that they were in use.

### 2.2. Statistical Methods

Data were collected from each participating center and transferred to RHCC after de-identification, where all analyses were performed. Categorical variables were compared using the χ^2^ or Fisher’s exact test, as appropriate, and continuous variables using Student’s *t*-test for normally distributed variables and the Mann–Whitney U test where distributions were non-normal. Variables were selected for inclusion in the multivariable logistic regression model based on statistical significance in the univariate analysis (*p* < 0.05), as well as clinical relevance and their potential association with mortality, to identify factors independently associated with 30-day mortality. Collinearity among candidate predictors was assessed using variance inflation factors, and no substantial collinearity was detected. Linearity of the logit was examined for continuous predictors. Repeat candidemia episodes in the same patient were treated as independent observations. Missing data were not imputed; each analysis included all available observations for the relevant variables (complete-case analysis). A two-sided *p*-value < 0.05 was considered statistically significant.

In mixed-species episodes, the first *Candida* species recovered from the blood culture, which was generally considered the predominant isolate, was used for the episode-level statistical analyses.

## 3. Results

### 3.1. Cohort General Characteristics and Risk Factors

During the study period, there were a total of 498 candidemia episodes among 482 pediatric patients across nine hospitals; 55.3% of episodes occurred in males. Most patients (98.8%) experienced a single episode, while six patients had recurrent episodes during the study period, two patients had two episodes, two had three episodes one had four episodes, and one had eight episodes. The mean duration of candidemia across the cohort was 4.2 days, with a median duration of 2 days (range, 1–35 days). Nine candidemia episodes involved infections with more than one *Candida* species.

Eighty-two patients (16.5%) had exposure to antifungal treatment within the two weeks preceding the episode of candidemia, of whom 58 (70.7%) received fluconazole.

Main demographic and clinical characteristics and 30-day univariate regression analysis are presented in Table 1.

### 3.2. Candida Susceptibility Patterns

**Figure 1:**
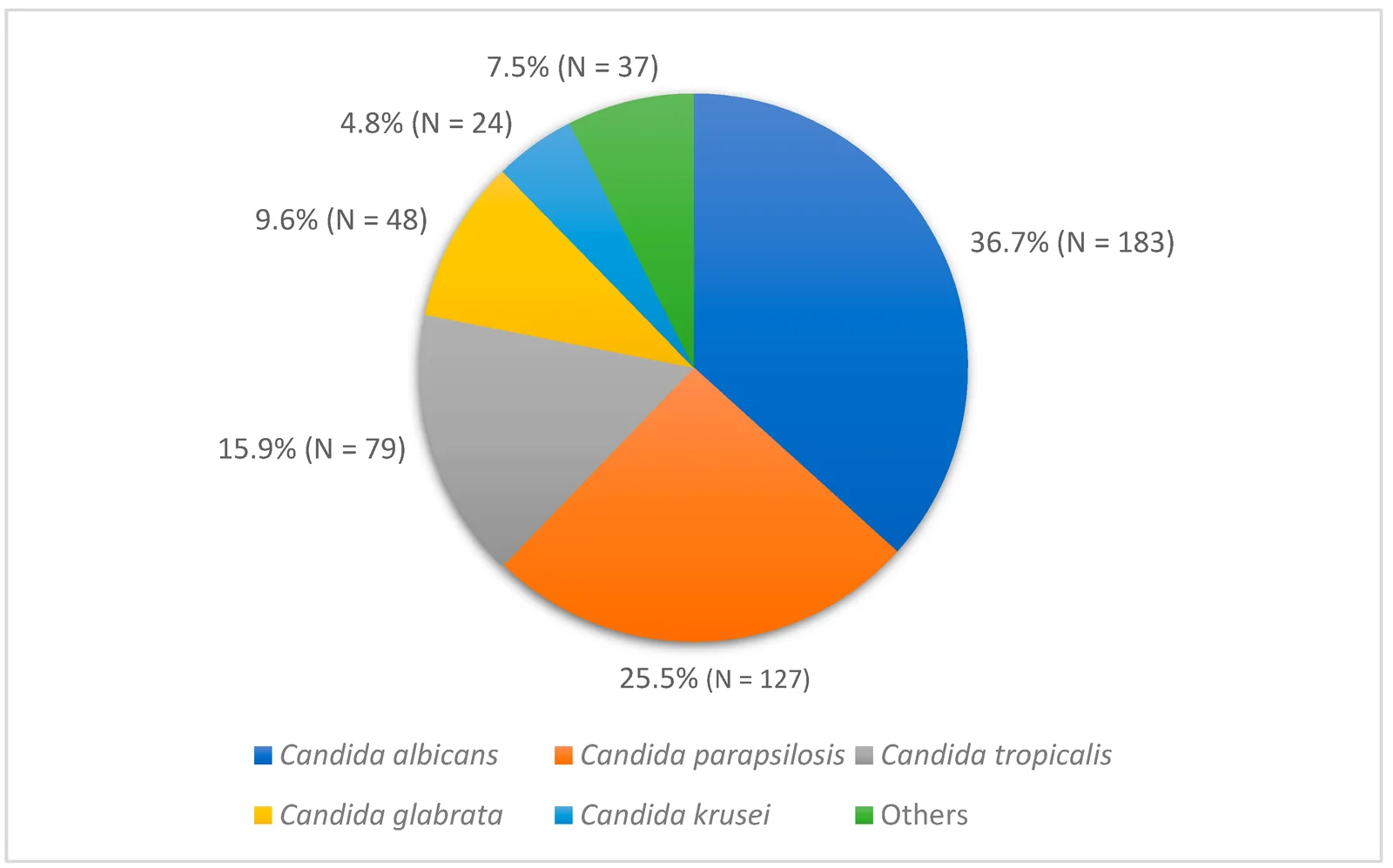
*Candida* isolates distribution.

Non-*albicans* species were more common than *C. albicans* (315 episodes (63.3%) vs. 183 episodes (36.7%)). Among the non-*albicans* isolates, *C. parapsilosis* was the most common species (n = 127, 25.5%), followed by *C. tropicalis* (n = 79, 15.9%), *C. glabrata* (*Nakaseomyces glabratus*) (n = 48, 9.6%), and *C. krusei* (*Pichia kudriavzevii*) (n = 24, 4.8%) (Figure 1). No episodes of *C. auris* were documented during the study period. The isolates grouped as “Other” in Figure 1 comprised *C. lusitaniae (Clavispora lusitaniae)* (n = 12), *C. dubliniensis* (n = 4), *C. guilliermondii* (n = 3), *C. pelliculosa (Wickerhamomyces anomalus)* (n = 3), *C. rugosa* (n = 2), *C. utilis* (n = 2), *C. kefyr* (n = 1), *C. orthopsilosis* (n = 1), *C. lipolytica* (n = 1), and *Cyberlindnera fabianii* (n = 1), together with seven isolates identified only to the genus level (*Candida* spp.). Most of these species lack established CLSI clinical breakpoints and were therefore not included in the fluconazole and caspofungin analyses.

Nine candidemia episodes involved mixed-species growth. To avoid counting a single candidemia episode more than once in the species-distribution analyses, only the first *Candida* species isolated in each episode was included in the primary species counts reported above. The additional species recovered in these mixed episodes were *C. glabrata* (n = 3), *C. tropicalis* (n = 3), *C. krusei* (n = 2), *C. albicans* (n = 1), *C. parapsilosis* (n = 1), and *Cyberlindnera fabianii* (n = 1). One episode involved three species (*C. albicans*, *C. krusei*, and *C. glabrata*), while the remaining eight involved two species. 

A comparison between *C. albicans* and non-*albicans candida* (NAC) using univariate analysis demonstrated differences mainly among immunocompromised patients (Table 2).

**Figure 2:**
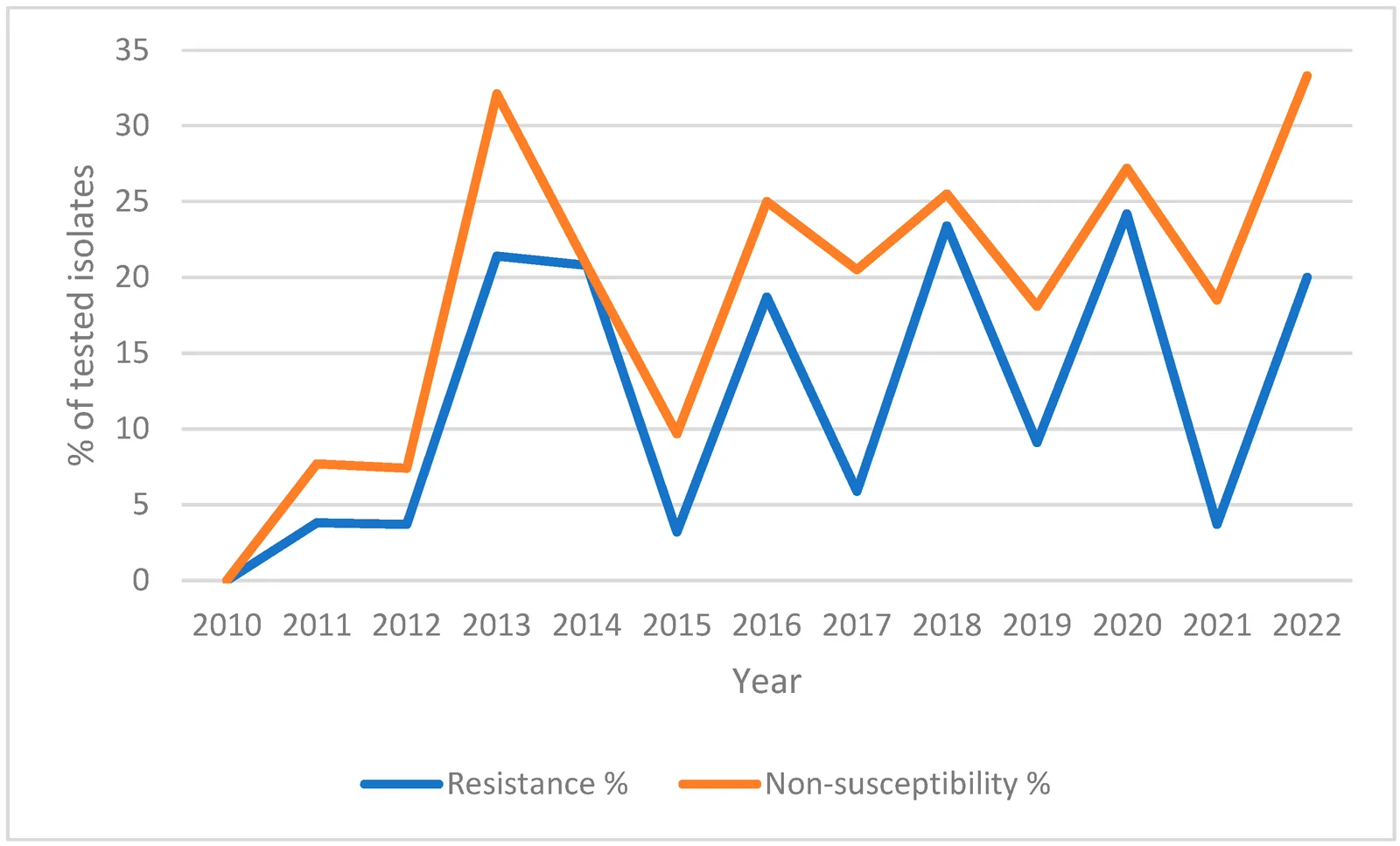
Fluconazole resistance and non-susceptibility over the years.

Fluconazole susceptibility was available for 376 isolates (the remaining 122 episodes had no defined breakpoints for the specific *Candida* species or missing susceptibility results). Of these, 303 (80.4%) were found to be sensitive, 25 (6.6%) were susceptible-dose dependent (SDD), and 48 (12.7%) were resistant. All SDD and resistant isolates were non-*albicans* species, with resistance observed mainly in *C. parapsilosis* and *C. glabrata*, and SDD primarily in *C. glabrata*. Resistance rates increased from 10.6% between 2010 and 2016 to 14.8% between 2016 and 2022 (OR 3.11, 95% CI 2.15–8.71, *p* = 0.015) and Non-susceptibility (SDD and resistant) rates increased from 15.5% between 2010 and 2015 to 23.3% from 2016 to 2022 (OR 2.89, 95% CI 1.45–5.11, *p* = 0.007) (Figure 2).

Caspofungin Susceptibility: 301 isolates had MIC results, 256 isolates (85%) were sensitive, 12 (4%) were resistant (6 were *C. parapsilosis*, 4 *C. krusei* and 2 *C. glabrata*), and 33 (11%) demonstrated intermediate susceptibility (10 were *C. parapsilosis*, 9 *C. glabrata*, 7 *C. tropicalis*, 4 *C. albicans*, and 3 *C. krusei*). Susceptibility patterns to caspofungin were stable through the study period. Susceptibility data were unavailable for 172 isolates, while the remaining isolates lacked defined CLSI breakpoints.

Amphotericin B susceptibility data were available for 307 episodes, of which only 4 (1.3%) were resistant: 2 *C. glabrata* and 2 *C. krusei*.

### 3.3. Candida Source or Organ Involvement

Most of the episodes were related to central lines (355; 71.2%) and were defined as central line-associated bloodstream infection (CLABSI). Twenty percent (102 episodes) were classified as candidemia without a defined source; among these episodes, 39 (38.2%) patients received total parenteral nutrition (TPN), 34 (33.3%) had a recent episode of bacteremia, 19 (18.6%) were neutropenic, and 30 (29.4%) were receiving corticosteroid therapy.

Sites and manifestations of *Candida* involvement included the urinary tract (n = 48, 9.6%), skin lesions (n = 33, 6.6%), peritonitis (n = 28, 5.6%), and disseminated candidiasis (n = 23, 4.6%), endocarditis (n = 14, 2.8%), endophthalmitis (n = 7, 1.4%), meningitis (n = 5, 1.0%), brain abscesses (n = 4, 0.8%), esophagitis (n = 2, 0.4%), and osteomyelitis/septic arthritis (n = 1, 0.2%). Some patients had more than one organ involved.

In cases with CLABSI, the line was removed in 220 episodes (61.9%). While in 95 episodes (26.7%) it was reported that the line was not removed. In these episodes, it is unclear whether the line was retained due to rapid clinical deterioration and death or because of vascular access limitations, as this information could not be determined given the retrospective nature of the study. In 33 episodes, data regarding line status and removal were unavailable.

### 3.4. Management

Regarding primary antifungal therapy, 166 patients (33.3%) received fluconazole, 156 (31.3%) were treated with amphotericin B formulations, 82 (16.5%) received an echinocandin, and 18 (3.6%) received voriconazole. In 76 episodes (15.3%), data on empiric treatment were either missing or the patient did not receive antifungal therapy due to early death before *Candida* isolation.

In most episodes, antifungal therapy was continued or adjusted based on susceptibility testing results. Treatment was continued with fluconazole in 165 patients (33.1%), amphotericin B formulations in 50 (10%), echinocandins in 60 (12.0%), and voriconazole in 21 (4.2%). The remaining patients either died before susceptibility results were available or had missing data.

### 3.5. Complications and Mortality

Candidemia was associated with significant clinical complications. Shock was reported in 91 patients (18.3%), respiratory failure in 78 patients (15.7%), and renal failure in 43 patients (8.6%).

Thirty days’ mortality was 23.4% (113 children) among the cohort, mean age 4.04 years, median 1.43 years, and 51.3% were male. Multivariate logistic regression analysis identified several factors independently associated with 30-day mortality (Table 3). Significant predictors of increased mortality included liver disease, neutropenia, TPN use, urinary tract infection, skin lesions, and peritonitis. Meningitis, which almost approached statistical significance demonstrated a high odds ratio (OR 13.77, 95% CI 1.03–15.88; *p* = 0.051), suggesting a possible association with increased mortality.

Regarding central line removal in CLABSI patients, mortality was significantly lower in univariate analysis in patients who underwent line removal compared with those who did not (15.3% vs. 39.6%, *p* < 0.001). However, this association was not demonstrated in the multivariate regression analysis (*p* = 0.057).

## 4. Discussion

This multicenter study describes the clinical characteristics, epidemiology, antifungal resistance patterns, and mortality risk factors of candidemia in pediatric patients over the past decade.

Our findings showed that non-*albicans Candida* dominated in the last 13 years, comprising 63.3% of the total episodes, while *C. albicans* is still the most common *Candida* species isolated (36.7%), and *C. parapsilosis* (25.5%) is the most isolated from the non-*albicans* group. Similar results were also observed in other pediatric studies [10,15,16,17,18].

A comparison between patients with *C. albicans* and non-*albicans Candida* showed dominance of non-*albicans* among immunocompromised patients; the reason may be related to long stays in hospital and previous antifungal treatment [10,15]. A study conducted in Israel reviewed the cases of pediatric C. *lusitaniae* and showed that a few cases, mainly among immunocompromised patients, were sensitive to fluconazole and had low mortality [19].

*C. auris* is a multidrug-resistant *Candida* species that has emerged over the past decade and is associated with significant global concern [8,20]; no episodes of *C. auris* were isolated throughout the entire study period.

None of the *Candida albicans* isolates in our cohort were resistant to fluconazole. Overall, fluconazole resistance was recorded in 12.7% of isolates, predominantly among *C. parapsilosis* and *C. glabrata*, which is consistent with rates reported in previous studies [2,17]. Korem et al. also reported high rates (40%) of fluconazole-resistant *C. parapsilosis* in Israel, attributing this finding to the nosocomial spread of hospital-specific clones harboring the Y132F mutation [21].

It is important to note that the definition of fluconazole resistance varies across studies. Some studies define resistance more broadly as non-susceptibility, referring to MIC values above the established susceptibility breakpoints. When applying this definition, our study shows 19.4% non-susceptibility to fluconazole, with a rise from 15.5% between 2010 and 2016 to 23.3% from 2017 to 2022. Rokkedal Lausch et al. reported an overall resistance rate of 12%, with an increase from 7.8% in 2004–2009 to 17.7% in 2010–2014 (*p* = 0.051). Fluconazole resistance was most prevalent among older children, rising from 8.8% to 33.3% during the study period (*p* = 0.023) [1]. Onal et al. reported overall 21.5% resistance to fluconazole in all *Candida* isolates, with 32.7% resistance to fluconazole in non-*albicans* isolates and 0% in *C. albicans* [22]. Piqueras et al. reported lower resistance levels, with only 3.3% of *Candida* isolates being resistant to fluconazole, with no increase over 2006–2016 [18]. Blyth et al. reported fluconazole resistance in 5% of *Candida* isolates, with a notably higher rate of SDD isolates in neonates (16.7%) compared to older children (1.2%) [23].

Caspofungin resistance was reported in 4% of our isolates with available sensitivity; six were *C. parapsilosis*, four were *C. krusei* and two were *C. glabrata*, and 11% demonstrated intermediate susceptibility. Benedict et al. reported only three (≈1%) episodes of echinocandin resistance out of 307 *Candida* isolates [17]. Pemán et al. reported only one (0.5%) *C. tropicalis* isolate that was resistant out of 203 episodes of candidemia [24]. Karadag-Oncel et al. reported only two (0.8%) *C. parapsilosis* isolates resistant to echinocandins out of 248 episodes [25]. Another Israeli study demonstrated 7.5% resistance [10], while Onal et al. reported 15% resistance to echinocandins, but without stating if they also included isolates with intermediate MIC in the resistance group [22]. Monitoring echinocandin resistance in pediatric populations is essential, given the high prevalence of *C. parapsilosis* and the use of echinocandins as first-line empiric therapy.

The 30-day mortality in our cohort was 23.4%, closely aligning with the 19.7% reported in another pediatric study from a tertiary care center in Israel [10]. Other studies reported lower mortality rates (10.2–17.2%) [1,17,26]. This difference may be explained by the higher proportion of patients in our cohort who were admitted to the PICU (27.4%), suggesting more severe or complex disease. In addition, most episodes occurred in tertiary centers, which may partly account for the higher mortality, as these centers typically manage more critically ill patients. Notably, the patients who died were very young, with a median age of less than 2 years.

Multivariate logistic regression analysis identified several factors independently associated with 30-day mortality, liver disease, neutropenia, TPN, urinary tract infection, skin lesions, and peritonitis.

Liver disease has also been reported in a limited number of studies as a risk factor for mortality. Silvester et al. reported that liver disease was significantly associated with mortality in multivariate regression analysis (*p* = 0.03) [2]. Bartoletti et al. reported that acute-on-chronic liver failure is a risk factor for candidemia, though without specifying its association with mortality (relative risk ratio 2.22, *p* = 0.046) [27].

Neutropenia is a well-recognized risk factor for mortality in adult patients with candidemia. In the pediatric population, however, this association has been less consistently demonstrated. To our knowledge, only one previous pediatric study reported an association between neutropenia and mortality in univariate analysis, which was not retained in the multivariable analysis [28]. In adults, previous studies have demonstrated an association between neutropenia and mortality, particularly in patients with persistent neutropenia, such as following induction therapy for hematologic malignancies [29,30,31].

TPN is also a well-established risk factor for candidemia in adult populations [32,33]. Korem et al. reported it as a risk factor for mortality in pediatric patients in univariate analysis (*p* = 0.024) but not in a multivariate analysis [10]. Kutlo et al., in a large multicenter prospective cohort study, reported TPN as an independent predictor of increased 30-day mortality in adult candidemia patients, with an OR of 3.9 (95% CI: 1.75–8.81, *p* = 0.001) after adjustment for other risk factors [32]. Andes et al. reported higher mortality and failure rates in adult patients receiving TPN (*p* = 0.004 and *p* = 0.002, respectively) [33].

Meningitis, peritonitis, and skin lesions have been identified as risk factors for mortality in our cohort; this may be reflecting disseminated or severe systemic disease among immunocompromised patients. Urinary tract infection has also been reported as a risk factor for mortality; this may reflect the need for urinary catheterization in these patients, which often indicates a more severe and debilitated clinical status [34].

Our study demonstrated lower mortality among patients who underwent central line removal (15.3% vs. 39.6%). However, this association was not maintained in the multivariate analysis. The current guidelines of the Infectious Diseases Society of America (IDSA), as well as the most recent 2025 guidelines published by the European Confederation of Medical Mycology (ECMM) in collaboration with the International Society for Human and Animal Mycology (ISHAM) and the American Society for Microbiology (ASM), recommend line removal as a key step in the management of candidemia, and to be performed as early as possible [11,35].

A patient-level quantitative review of seven randomized trials by Andes et al. found that central line removal was independently associated with decreased mortality (OR = 0.50, 95% CI 0.35–0.72) and improved clinical success, even after adjusting for severity of illness and other confounders [33]. Karadag-Oncel et al. reported that failure to remove the line was associated with a 20.5-fold increase in mortality [95% CI: 3.9–106.5, *p* < 0.001] in infants younger than 3 months, and a 23-fold increase [95% CI: 7.48–70.77, *p* < 0.001] in patients older than 3 months [25]. In contrast, a study of 842 adult patients with candidemia found no clinical benefit of early central line removal [36]. Our study results warrant further analysis to clarify the exact impact of early line removal on outcomes.

## 5. Study Limitations

Our study has several limitations. First, its retrospective design limited the ability to collect certain data during the episodes, and some information was missing and could not be retrieved; however, the large number of episodes may have helped overcome this gap. Second, susceptibility data were missing for a considerable proportion of isolates, restricting the comprehensiveness of antifungal resistance assessment. In addition, microbiological methods were not standardized across participating centers and changed over the long study period. Detailed information regarding the specific systems, database versions, and identification thresholds used at each center and time point could not be retrospectively retrieved. These methodological variations may have influenced susceptibility results, and observed temporal trends. Finally, variability in clinical practice and diagnostic criteria across participating centers may have introduced heterogeneity in data interpretation. The large cohort may overcome some of the limitations and the fact that the known risk factors were found may add to that.

## 6. Conclusions

Pediatric candidemia remains a severe infection with high morbidity and substantial mortality. Non-*albicans* species are becoming more frequent than *C. albicans*, with *C. parapsilosis* predominating, while no episodes of *C. auris* were identified. Over the past decade, a significant increase in fluconazole non-susceptibility was observed. Mortality was strongly associated with liver disease, neutropenia, TPN use, and deep-seated infections. Further research is needed in pediatrics due to rising *Candida* resistance worldwide and the rising numbers of critically ill patients.

## Figures and Tables

**Table 1:** Demographic, clinical characteristics and 30-day univariate analysis.

		All (n = 498)	30-Day Mortality—No (n = 385)	30-Day Mortality—Yes (n = 113)	*p*-Value
Age (years)	Mean	3.66	3.59	4.04	0.585
Admission Department	General pediatrics	103 (20.7%)	95 (24.7%)	8 (7.1%)	
	Hemato-oncology	104 (20.9%)	85 (22.1%)	19 (16.8%)	0.223
	PICU	138 (27.7%)	91 (23.6%)	47 (41.6%)	<0.0001
	NICU	95 (19.1%)	68 (17.7%)	27 (23.9%)	0.157
	Surgery/Cardiology	57 (11.4%)	45 (11.7%)	12 (10.6%)	0.700
Main diagnosis/disease group	Hemato-oncologic/other immune deficiency	106 (21.3%)	73 (19.1%)	33 (29.2%)	0.276
	Cardiac/pulmonary	94 (18.9%)	64 (16.6%)	30 (26.5%)	0.01
	Renal/metabolic	38 (7.6%)	31 (8.1%)	7 (6.2%)	0.462
	Liver/Gastrointestinal	69 (13.9%)	61 (15.8%)	8 (7.1%)	0.015
	Trauma/dermatologic	18 (3.6%)	15 (3.9%)	3 (2.7%)	0.535
	Neuro/neurosurgical	13 (2.6%)	12 (3.1%)	1 (0.9%)	0.15
Received systemic steroids		123 (24.7%)	89 (23.1%)	34 (30.1%)	0.102
Received TPN (in the last month)		219 (44.0%) *	159 (41.3%)	60 (53.1%)	0.02
Received antifungal treatment (in the last month)		82 (16.5%)	53 (13.8%)	29 (25.7%)	<0.001
Severe neutropenia (ANC < 500)		124 (24.9%)	83 (21.6%)	41 (36.3%)	0.003
Prematurity		122 (24.5%)	94 (24.4%)	28 (24.8%)	0.94
Transplant patients	HSCT	25 (5.0%)	17 (4.4%)	8 (7.1%)	0.189
	solid organ	1 (0.2%)	1 (0.3%)	0 (0%)	0.403
Chronic gastrointestinal disease		83 (16.7%)	73 (19.0%)	10 (8.8%)	0.011
Liver disease		26 (5.2%)	19 (4.9%)	7 (6.2%)	0.03
Chronic renal disease		28 (5.6%)	20 (5.2%)	8 (7.1%)	0.50
Diabetes		2 (0.4%)	2 (0.6%)	0 (0%)	1
Recent surgery		91 (18.3%)	62 (16.1%)	29 (25.7%)	0.02
Bacteremia during the last month		221 (44.4%)	166 (43.1%)	55 (48.7%)	0.935

* Data were unavailable for 25 patients. TPN—total parenteral nutrition, ANC—absolute neutrophil count, HSCT—hematopoietic stem cell transplant.

**Table 2:** Comparison between *C. albicans* and non-*albicans Candida* (NAC) patients.

	*C. albicans* (n = 183)	Non-*Albicans* (n = 315)	*p*-Value
Sex (Male)	103 (56%)	174 (55%)	
Age (Mean)	3.10	3.99	
Previous episode of candidemia	11 (6%)	16 (5.1%)	0.68
Prematurity	41 (22.4%)	81 (25.7%)	0.49
Bone marrow transplant	5 (2.73%)	23 (7.30%)	**0.04**
Solid organ transplant	2 (1.1%)	7 (2.2%)	0.50
Diabetes	1 (0.5%)	1 (0.3%)	1.00
Chronic gastrointestinal disease	36 (19.7%)	47 (14.9%)	0.20
Liver disease	13 (7.1%)	13 (4.1%)	0.21
Chronic renal disease	7 (3.8%)	21 (6.7%)	0.27
Neutropenia (ANC < 500)	36 (19.6%)	88 (27.9%)	**0.05**
Total parenteral nutrition (TPN)	86 (47%)	133 (42.2%)	0.32
Recent bacteremia	87 (47.5%)	134 (42.5%)	0.30
Meningitis	2 (1.1%)	3 (0.1%)	1.00
Urinary tract infection	25 (13.7%)	23 (7.3%)	**0.03**
Skin lesions	12 (6.6%)	21 (6.6%)	1.00
Peritonitis	14 (7.7%)	14 (4.4%)	0.19
Line removal	81 (44.3%)	138 (43.8%)	0.96
Immunodeficiency	44 (24%)	104 (33%)	**0.048**

**Table 3:** Thirty-day mortality regression analysis.

	Odds Ratio	*p*-Value	95% CI
Sex (Male)	1.17	0.616	0.71–1.81
Age	0.98	0.434	0.94–1.02
Previous episode of candidemia	0.61	0.461	0.13–2
Prematurity	0.98	0.956	0.53–1.78
Bone marrow transplant	1.25	0.623	0.50–2.98
Solid organ transplant	0.13	0.091	0.01–1.03
Diabetes	0.15	0.080	0.11–1.12
Chronic gastrointestinal disease	0.92	0.151	0.10–1.39
Liver disease	3.10	**0.031**	1.07–8.58
Chronic renal disease	1.15	0.446	0.46–4.12
Neutropenia (ANC < 500)	2.23	**0.004**	1.29–3.87
Total parenteral nutrition (TPN)	2.17	**0.002**	1.33–3.58
Recent bacteremia	1.58	0.058	0.99–2.54
*Candida* species (*Albicans* vs. non-*albicans*)	0.91	0.720	0.56–1.48
Meningitis	13.77	0.051	1.03–15.88
Urinary tract infection	2.81	**0.005**	1.35–5.78
Skin lesions	2.56	**0.023**	1.13–5.75
Peritonitis	5.77	**0.000**	2.29–15.21
Line removal	0.82	0.057	0.80–3.15

ANC—absolute neutrophil count.

## Data Availability

The data presented in this study are available from the corresponding author upon reasonable request.
